# Endoscopic decompression of a C1 osteophyte causing bow hunter’s syndrome in a 22-year-old male

**DOI:** 10.3171/2024.1.FOCVID23234

**Published:** 2024-04-01

**Authors:** Zachary A. Abecassis, John I. Ogunlade, Whitney Teagle, Guilherme Barros, Christine Park, Michael R. Levitt, Christoph P. Hofstetter

**Affiliations:** 1 Department of Neurological Surgery, University of Washington, Seattle, Washington; and; 2 Department of Neurological Surgery, Washington University School of Medicine in St. Louis, Missouri

**Keywords:** endoscopic spine, vertebral artery compression, bow hunter’s syndrome

## Abstract

The patient is a 22-year-old male with a history of C1 avulsion fracture causing vertebral artery compression with pseudoaneurysm and symptomatic stroke. Cerebral angiography demonstrated dynamic compression of the V3 segment of the vertebral artery due to a chronic C1 avulsion fracture. The authors utilized a full endoscopic approach with intraoperative angiography for proximal control and Doppler ultrasound to confirm adequate decompression. The surgery duration was 3 hours with blood loss < 5 ml. The patient was discharged on postoperative day 1 with no complication and has been asymptomatic since surgery. This is the first documented use of endoscopic decompression to treat this condition.

The video can be found here: https://stream.cadmore.media/r10.3171/2024.1.FOCVID23234

## Transcript

### 0:41

The patient is a 22-year-old with a history of a left C1 avulsion fracture who presented with dizziness and visual changes. He was found to have multiple infarcts on MRI and a CTA showed a C1 osteophyte impinging the left vertebral artery causing a pseudoaneurysm. Despite anticoagulation he had progression of his strokes and remained symptomatic. The patient underwent dynamic cerebral angiogram which showed non–flow-limiting stenosis at the level of C1–2 associated with a protruding chronic C1 avulsion fracture of C1.^1–4^

### 1:08

The patient was placed prone in three-point fixation with a Mayfield head holder with the sheath protected in the event of a vertebral artery hemorrhage.

First, the puncture site was confirmed using fluoroscopy in the anteroposterior and lateral view confirming our entrance at the C1–2 joint. We chose the skin incision based on approximately a 45° angle to the lateral aspect of the joint, which was approximately 2–3 cm off the midline. This was localized with a spinal needle. We performed a 7-mm cross section with opening of the fascia and insertion of the dilator and working cannula, always watching for bone contact with the lateral edge of the bone window or the lateral edge of the facet joint.

### 1:48

Via a small skin incision, serial dilators were advanced and a dilator was advanced onto the lateral atlantoaxial joint. A tubular retractor was placed and a laminoscope, which was 7.3 mm in diameter, was brought in. Under direct visualization, soft tissue was dissected off the C1–2 joint using the bipolar cautery and the micropunch rongeur.

### 2:08

Once the lateral atlantoaxial joint was exposed, we visualized the protruding traumatic C1 osteophyte. Using a 3.5-mm diamond burr, the lateral aspect of the C1 osteophyte was resected. Once the lateral surface of the C1 lateral mass was flush with lateral surface of C1, we carefully utilized a Kerrison rongeur to decompress the vertebral artery.

### 2:38

Paying close attention to the vertebral artery, the micropunch was used to remove any tissue and remaining fragments encroaching on the vessel.

### 2:48

Good flow of the vertebral artery was confirmed with an intraoperative Doppler.

### 3:02

A postoperative cerebral angiogram was obtained through his radial access showing the left vertebral artery with a similar appearance of the known cervical dissection. There was interval removal of the bone spur from the inferior aspect of the C1 lateral mass. The patient was discharged from the hospital the following day without any complication. He returned to clinic 3 months after his procedure and his CTA showed a stable, resolving pseudoaneurysm with excellent decompression of the osteophyte.^5^

## Disclosures

Dr. Levitt reported grants from Medtronic and Stryker; equity interest from Proprio, Stroke Diagnostics, Synchron, Hyperion Surgical, and Apertur; financial interest from Fluid Biomed; being an adviser for Aeaean Advisers and a consultant for Stereotaxis and Metis Innovative; serving on the editorial boards of the *Journal of NeuroInterventional Surgery* and *Frontiers in Surgery*; and serving on the data and safety monitoring board for Arsenal Medical, outside the submitted work. Dr. Hofstetter reported being a consultant for Globus, Innovasis, and joimax; and teaching for AO Spine and Espinea.

## Author Contributions

Primary surgeon: Levitt. Assistant surgeon: Abecassis, Barros. Editing and drafting the video and abstract: Park, Abecassis, Ogunlade, Teagle, Barros. Critically revising the work: Abecassis, Ogunlade, Teagle, Barros, Levitt, Hofstetter. Reviewed submitted version of the work: all authors. Approved the final version of the work on behalf of all authors: Park. Supervision: Hofstetter.

## Supplemental Information

### Patient Informed Consent

The necessary patient informed consent was obtained in this study.
